# Dried Blood Spot-Based Monitoring of Dietary Treatment in Children, Adolescents, and Young Adults with Inherited Disorders of Amino Acid Metabolism: A Four-Year Pilot Study

**DOI:** 10.3390/nu18183020

**Published:** 2026-09-16

**Authors:** Agnieszka Kowalik, Ewa Głąb-Jabłońska, Joanna Taybert, Marta Nogalska, Mariola Sasin-Rokita, Magdalena Chełchowska, Joanna Myszkowska-Ryciak, Jolanta Sykut-Cegielska

**Affiliations:** 1 Department of Inborn Errors of Metabolism and Pediatrics, Institute of Mother and Child, 01-211 Warsaw, Poland; agnieszka.kowalik@imid.med.pl (A.K.);; 2 Department of Metabolic Screening and Diagnostics, Institute of Mother and Child, 01-211 Warsaw, Poland; 3 Department of Dietetics, Institute of Human Nutrition Sciences, Warsaw University of Life Sciences (WULS), 02-776 Warsaw, Poland

**Keywords:** inborn errors of metabolism, dietary treatment monitoring, dried blood spot, tandem mass spectrometry, amino acid metabolism disorders, metabolic control

## Abstract

**Background:** Regular biochemical monitoring is essential for the safe and effective dietary treatment of inherited disorders of amino acid metabolism. However, conventional venous blood sampling may limit monitoring frequency and increase the burden on families. This study evaluated the feasibility and clinical usefulness of home-collected dried blood spot (DBS) samples analyzed using tandem mass spectrometry and additional chromatographic methods for dietary monitoring in inherited disorders of amino acid metabolism other than phenylketonuria. **Methods**: Between 2022 and 2025, children, adolescents, and young adults aged between 4 months and 20.5 years who were followed at a national metabolic center were monitored using DBS samples. The initial cohort included patients with maple syrup urine disease, tyrosinemia type III, homocystinuria, glutaric aciduria type 1, methylmalonic aciduria, isovaleric aciduria, argininosuccinic aciduria, methionine adenosyltransferase I/III deficiency, beta-ketothiolase deficiency, propionic aciduria, gyrate atrophy, and citrullinemia type 1. Disease-specific amino acids and metabolites were assessed to guide individualized dietary recommendations. **Results**: The number of monitored patients ranged from 24 to 40 per year. Overall annual completion rates of planned DBS measurements ranged from 56.7% to 73.0%, with additional clinically indicated samples collected in selected patients. DBS monitoring detected abnormal metabolite concentrations and supported dietary interventions, particularly in maple syrup urine disease, glutaric aciduria type 1, homocystinuria, tyrosinemia type III, and MAT I/III deficiency. In several other disorders, routine monthly DBS monitoring showed limited additional clinical value. **Conclusions**: DBS-based monitoring is a feasible approach that may support individualized dietary management in selected inherited disorders of amino acid metabolism, especially in early childhood and during periods of increased metabolic risk.

## 1. Introduction

The development of the bacterial inhibition assay by the American microbiologist Robert Guthrie in 1960, designed to detect phenylketonuria using dried blood spot (DBS) samples, led to the implementation of newborn screening programs in many countries [1]. This approach transformed the diagnosis of phenylketonuria (PKU), enabled early dietary intervention, and subsequently paved the way for the use of DBS sampling in long-term biochemical monitoring.

DBS sampling is minimally invasive, simple to perform, and enables rapid and safe shipment of samples for laboratory analysis. These advantages make DBS a practical tool not only for newborn screening, but also for biochemical monitoring in patients requiring long-term dietary treatment. In PKU, DBS-based monitoring is well established and has become an integral component of dietary management, allowing regular assessment of blood phenylalanine concentrations and timely adjustment of treatment. Research on a faster, patient-centered system for home blood phenylalanine (Phe) testing has been ongoing for several years. Strong point-of-care testing (POCT) compliance with DBS and high family acceptance have been demonstrated, paving the way for transforming PKU care [2].

Regular biochemical monitoring is also essential for the dietary management of other inherited disorders of amino acid metabolism, including maple syrup urine disease (MSUD) [3], glutaric aciduria type 1 (GA1) [4], homocystinuria (HCU) [5], and selected forms of tyrosinemia [6]. According to current guidelines, disease-specific amino acids and metabolites, particularly during infancy and early childhood, should be monitored frequently, often at least once monthly. However, until recently, monitoring at many medical centers, including the Institute of Mother and Child in Warsaw, Poland, relied primarily on venous blood sampling performed during hospital or outpatient visits. This model may limit the frequency of follow-up, increase the burden on families, and delay dietary modifications when biochemical abnormalities occur between scheduled visits.

The use of MS/MS, together with supplementary column-separation methods, enables the measurement of amino acids and other disease-specific metabolites in DBS samples. In particular, column-based separation enables more precise assessment of branched-chain amino acids (BCAAs), which is essential in the monitoring of MSUD. This approach may therefore extend the benefits of DBS-based monitoring beyond PKU, enabling more frequent biochemical follow-up and individualized dietary intervention without the need for hospital-based blood collection.

Although DBS-based monitoring is well established in PKU [7], its use for long-term dietary monitoring in other inherited disorders of amino acid metabolism remains less widely implemented and requires further evaluation [8,9]. Therefore, this four-year pilot study aimed to assess the feasibility and clinical utility of DBS-based monitoring using tandem mass spectrometry and supplementary chromatographic methods in patients with inherited disorders of amino acid metabolism other than PKU followed at the Institute of Mother and Child in Warsaw, Poland.

## 2. Materials and Methods

### 2.1. Materials

Between 2022 and 2025, the study included 46 unique patients aged between 4 months and 20.5 years (29 males and 17 females) who were under the care of the Department of Inborn Errors of Metabolism and Paediatrics and the Metabolic Outpatient Clinic at the Institute of Mother and Child in Warsaw. The annual numbers of participants were 24 in 2022, 40 in 2023, and 25 in both 2024 and 2025. Phase 1 was conducted in 2022–2023, whereas Phase 2 was conducted in 2024–2025. The characteristics of the study groups in each year are presented in Table 1.

The following conditions were included: MSUD, tyrosinemia type III (TYR type III), methylmalonic aciduria (MMA), isovaleric aciduria (IVA), argininosuccinic aciduria (ASA), methionine adenosyltransferase I/III deficiency (MAT I/III deficiency), beta-ketothiolase deficiency (BKT deficiency), propionic aciduria (PA), gyrate atrophy, and citrullinemia type 1 (phase 1). The enrollment of patients with HCU and GA1 commenced in 2023.

In phase 2, patients with MSUD, TYR type III, HCU, and GA1 were retained. Conversely, patients with MMA, IVA, ASA, MAT I/III deficiency, BKT deficiency, PA, gyrate atrophy, and citrullinemia type 1 were withdrawn from further DBS-based monitoring in 2024 after two years of dietary treatment monitoring using this method, because no abnormalities in the relevant amino acid concentrations and no need for dietary intervention had been identified. Nevertheless, patients with the aforementioned inherited metabolic disorders continued to undergo regular medical and laboratory follow-up, the frequency of which proved sufficient for monitoring their treatment.

Each parent received twelve labeled dried blood spot cards per year, with instructions to collect a blood sample on the card during the first few days of each month and send it to the laboratory. Parents and caregivers received practical training in DBS sample collection during an in-person visit, together with written instructions and access to an instructional video demonstrating the correct sampling procedure (available online: https://przesiew.imid.med.pl/pobieranie.html, accessed on 15 September 2026). Samples were collected at home. To ensure adequate sample quality, parents and caregivers were instructed not to touch the designated blood collection areas of the filter-paper card and to discard the first drop of blood. Each marked circle was filled completely from one side of the card, allowing the blood to soak through to the reverse side. Blood was not applied from both sides of the card or in multiple overlapping layers, and hematocrit capillary tubes were not used.

After collection, the filter-paper card was dried vertically at room temperature, away from direct sunlight and heat sources, for approximately two hours. Only completely dried cards were placed in paper mailing envelopes and transported to the laboratory; plastic bags and foil packaging were not used. Upon receipt, each sample was visually assessed for complete and uniform saturation of the required collection areas and for the absence of contamination, damage, multilayered blood application, or insufficient drying. Samples that did not meet these predefined quality requirements were considered unsuitable for analysis and were rejected. The results of the analyses, accompanied by recommendations regarding the continuation of the diet in its current state or the implementation of suitable dietary modifications, were communicated to the parents via email. In instances of atypical results pertaining to infection, metabolic deterioration, surgical interventions, or dietary imbalances, an additional dried blood spot card was submitted as soon as possible.

Patient inclusion criteria for the project were as follows:

• Confirmed diagnosis of an inherited metabolic disorder in the child;
• Parental/legal guardian consent for participation in the project for a 12-month period, renewed annually;
• Completion of training in the finger-prick dried blood spot sampling procedure;
• Consent to provide, by email, information regarding the child’s health status, body weight, and diet followed during the month preceding dried blood spot collection.

No specific exclusion criteria were applied in the study. Two eligible patients did not participate because their parents did not provide consent for inclusion in the project.

### 2.2. Methods

The tandem mass spectrometry method using flow injection analysis included the determination of two profiles: amino acids and carnitine esters. The analyses were performed on API 3500 mass spectrometers (AB SCIEX, Framingham, MA, USA). A laboratory-developed method, adapted from previously published analytical procedures, was used [10,11,12,13]. The procedures were modified according to the laboratory’s analytical requirements and internally validated before their implementation in routine analysis. The analyses were based on stable isotope-labeled internal standards and calibration curves. For each analyte, the abundance, expressed as signal intensity, of characteristic precursor and product ions was determined. The signal intensity measurements for selected precursor/product ion pairs were performed in multiple reaction monitoring (MRM) mode. The concentrations of metabolites were calculated from the ratio of the signal intensity of patient samples to that of the internal standards, in conjunction with the slope of the calibration curve.

Since leucine (Leu), isoleucine (Ile) and alloisoleucine (allo-Ile) possess identical mass profiles, and because lysine (Lys) and tryptophan (Trp) are not determined using the basic method, an additional column-based separation method was developed for branched-chain amino acids and the aforementioned amino acids. The experiment utilized isotope-labelled standards for Leu, Ile, valine (Val), and Lys, in conjunction with dedicated in-house calibration curves. The separation process was conducted using a Kinetex F5 chromatographic column (Phenomenex; 50 mm × 2 mm; particle size, 2.6 µm). The separation was performed using ion-pair chromatography on API 3200 and QTRAP 5500 mass spectrometers (AB SCIEX). Heptafluorobutyric acid (HFBA) was utilized as an ion-pairing reagent. The samples were then subjected to analysis in MRM mode. The concentrations were determined through the calculation of peak areas, with the analyte and the isotope-labelled internal standard being measured. The concentration ratio was subsequently calculated and referenced to the calibration curve.

In the fundamental MS/MS analysis of amino acid and carnitine ester profiles, the quantification of homocysteine is not possible, and the differentiation of PA from MMA is not feasible. Consequently, an additional column-based separation method employing DBS samples was implemented, thereby enabling the concurrent estimation of homocysteine (Hcy), methylmalonic acid (MMA), and methylcitric acid (MCA). The concentrations were determined through the calculation of peak areas, with the analyte and the isotope-labelled internal standard being measured. The concentration ratio was subsequently calculated and referenced to the calibration curve. The measurements were performed on a QTRAP 5500 + mass spectrometer (AB SCIEX, Framingham, MA, USA) using a Synergi Fusion-RP column (Phenomenex, Torrance, CA, USA; 50 mm × 2 mm; particle size, 4 µm). Blood homocysteine concentrations were converted by multiplying each result by 20. The conversion factor of 20 was derived from the approximately twentyfold difference between the reference values established in our laboratory for DBS LC-MS/MS and plasma HPLC measurements and was evaluated using paired DBS and plasma samples. This approach is also consistent with previous observations that homocysteine concentrations measured in DBS are lower than those measured in corresponding plasma samples [14].

Measuring amino acid concentrations in filter paper required reference to the target reference values for these amino acids in plasma for patients with inborn errors of metabolism. It was based on available reports concerning validation of plasma cut-off values for use with DBS measurements [15,16].

For the purpose of monitoring dietary treatment in specific inherited metabolic disorders, the reference values for blood amino acid concentrations derived from those published in the guidelines, as below:

- Reference range for tyrosine and phenylalanine in tyrosinemia type III as in tyrosinemia type I [6]:

Children <12 years: Tyr, 200–400 µmol/L; Phe, >50 µmol/L.

Adults: Tyr, <500 µmol/L; Phe, >50 µmol/L.

- Reference range for leucine, isoleucine, and valine, that is, branched-chain amino acids (BCAAs), in MSUD [17]:

Leucine: 75–200 µmol/L in children <5 years of age.

Leucine: 75–300 µmol/L in patients >5 years of age.

Isoleucine: 200–400 µmol/L.

Valine: 200–400 µmol/L.

- Reference range for lysine and tryptophan in glutaric aciduria type 1 [4]:

Values within the reference range were accepted, with an optimal lysine concentration of 80–120 µmol/L in children <6 years of age.

In patients >6 years of age, values within the physiological reference range were accepted.

The optimal tryptophan concentration was >20 µmol/L.

- Reference range for homocystinuria [5]:

For B6-responsive HCU in children and adults: tHcy < 50 µmol/L.

For B6-nonresponsive HCU in children and adults: tHcy < 100 µmol/L.

- Reference range for methionine in diet-treated MAT I/III deficiency [18]:

A methionine concentration of <500 µmol/L.

- Reference range for BCAAs in blood for inherited metabolic disorders such as MMA, PA, IVA, ASA, BKT deficiency, gyrate atrophy, and citrullinemia type 1 [19]:

For these disorders, the reference range for healthy individuals was adopted.

Valine: 79.5–210 µmol/L.

Leucine: 62.8–158 µmol/L.

Isoleucine: 23.8–74.8 µmol/L.

Because of the pilot nature of the study and the small, heterogeneous disease-specific groups, analyses were descriptive. Categorical variables are presented as counts and percentages, and continuous variables as ranges, where appropriate. No formal hypothesis testing was performed.

## 3. Results

During the four-year project, 24 children participated in stage 1 in 2022 and 40 children in 2023. In stage 2, 25 children participated in 2024 and 25 children in 2025. Across all groups of inherited metabolic disorders, the age range was from four months to 20.5 years.

A comprehensive four-year follow-up period, encompassing both phases 1 and 2, was completed for five children with MSUD; one additional child underwent liver transplantation after three years. Two patients with MSUD were monitored using this method for two years, from 2024 to 2025, during phase 2.

A boy with tyrosinemia type III was monitored over the course of four years. In the HCU group, three of five children were followed for three years, beginning with their inclusion in the project in 2023, when measurement of tHcy in dried blood spot samples became available. One child was monitored for 1 year only, whereas the youngest girl with HCU was monitored from 5 months of age for 2 years during phase 2.

In 2023, the monitoring of patients with glutaric aciduria type 1 (GA1) was initiated, and 12 patients were enrolled. In 2024, an additional newborn patient with GA1 was included, increasing the cohort to 13 patients. All patients were followed prospectively through 2024–2025.

During the 2-year observation period from 2022 to 2023, five children with methylmalonic aciduria participated in the study. In the isovaleric aciduria group, eight children participated in 2022 and seven in 2023. Phase 1 also included one boy with ASA, one boy with MAT I/III deficiency, and one boy with BKT deficiency. One patient with PA, one with gyrate atrophy, and one with citrullinemia type 1 remained under dried blood spot-based monitoring for 1 year.

With the exception of one patient with TYR type III in the 2022–2023 period, the number of dried blood spot cards returned for analysis was lower than the planned 12 cards per child per year. The overall completion rate for the scheduled measurements averaged approximately 72%, 72%, 57%, and 73% in consecutive years from 2022 to 2025, respectively (Table 2).

The proportion of received DBS samples that were unsuitable for analysis did not exceed 1%. In such cases, the parents were informed; however, a repeat DBS collection was not required. The subsequent sample was collected according to the planned monitoring schedule, at the beginning of the following month, approximately three weeks later. The average interval between home sample collection and arrival at the laboratory was 2–4 days, the interval to receive laboratory results was usually 1–4 days. Then within an additional 1–2 days the results were communicated to the family and, when required, dietary recommendations were implemented.

The dietary interventions implemented in response to abnormal amino acid concentrations measured in dried blood spot samples for each inherited metabolic disorder are presented in Table 3.

An abnormal result was defined as a DBS-derived concentration of a disease-specific amino acid or metabolite outside the adopted therapeutic/reference range for the respective disorder. Dietary recommendations were individualized for each patient. They were prepared according to the dietary principles applicable to the specific inherited metabolic disorder [3,4,5,6,17,18,19] and adjusted to the patient’s current biochemical results. When an abnormal result was identified, the dietary consultation included a renewed discussion of the recommendations, identification of potential causes of the abnormal value, and, when necessary, modification of the dietary composition. This could involve adjusting the amount of specialized medical formula and/or the proportion of natural protein in the diet.

In the MSUD group, the total number of interventions was highest in the first year of the study (2022) and decreased by approximately 53% in subsequent years. Among the four patients with HCU, three required interventions in 2023 and 2025. Dietary modifications were most frequently implemented for one patient, the youngest child. In the GA1 group, 75% of children had one abnormal blood lysine result requiring dietary intervention in 2023. In subsequent years, this applied to 50% and 54% of children in this group, respectively. The number of interventions increased from nine in 2023 to 21 in 2025; however, 30% of these interventions in 2025 concerned only one patient, the youngest child in the group.

In the patient with MAT I/III deficiency, the number of interventions decreased from three to one in the final year of the study. Abnormal concentrations of branched-chain amino acids (BCAAs), most decreased blood isoleucine and/or leucine concentrations, were found in one of five children with methylmalonic aciduria (MMA) during the two-year observation period. In contrast, no deviations from the reference ranges were observed for the relevant amino acid concentrations, including BCAAs, in children with IVA, ASA, BKT deficiency, PA, gyrate atrophy, and citrullinemia type 1.

## 4. Discussion

The standard of care for patients diagnosed with inborn errors of metabolism, including organic acidurias, disorders of amino acid metabolism, and fatty acid oxidation disorders, consists of regular clinical, biochemical, and dietary monitoring. More frequent biochemical monitoring may facilitate earlier detection of metabolic imbalance and allow timely dietary adjustment, particularly in disorders associated with a risk of metabolic decompensation. The ability to identify potential health- or life-threatening conditions, including metabolic decompensation or metabolic crises, is enhanced by the increased frequency of monitoring.

Optimal management of patients with inborn errors of metabolism requires regular assessment of clinical status, including medical history and physical examination; evaluation of disease-specific biochemical markers; and assessment of dietary management based on dietary history and 3-day food records. Until recently, this approach was performed mainly during visits to the Metabolic Outpatient Clinic or during hospitalization at the Department of Inborn Errors of Metabolism and Paediatrics of the Institute of Mother and Child.

The World Health Organization has acknowledged population-based newborn screening using DBS as a pivotal preventive strategy aimed at the early detection of inborn errors of metabolism and the prompt initiation of appropriate treatment. It is well established that delayed diagnosis, or the absence of treatment, may pose a threat to the child’s life or lead to impaired development, severe disease course, and/or permanent intellectual disability [20,21].

For many years, a DBS-based system for monitoring dietary treatment in phenylketonuria has demonstrated the effectiveness of this approach in achieving therapeutic targets during long-term follow-up. In a multicenter study encompassing over 1300 patients with PKU, while blood Phe control deteriorated with age, more frequent blood sampling was correlated with enhanced control of blood Phe concentrations and diminished variability [22].

Other disorders, such as MSUD and GA1, have been associated with a risk of metabolic decompensation and potential central nervous system injury. Consequently, particularly during the initial years of life, these conditions necessitate more frequent monitoring of amino acid concentrations to ensure stable metabolic control, as outlined in the available guidelines [3,4]. Concurrent monthly monitoring of amino acid concentrations in HCU, TYR type III, and diet-treated MAT I/III deficiency may reduce the risk of organ-related complications in these inherited metabolic disorders [5,6,18,23].

It was observed that the implementation of monthly monitoring of metabolic control using this method resulted in a significant increase in the frequency of modifications to dietary recommendations for infants and children when compared with the feasibility of modifications that would be possible with in-person monitoring, which requires the collection of venous blood for plasma amino acid analysis. This approach may, therefore, support better control and maintenance of metabolic stability, as well as stable somatic development in children. Current information regarding the child’s health status, body weight, and feeding difficulties, in conjunction with biochemical results, is consistently beneficial and imperative for customizing dietary modifications to the individual needs and clinical condition of the child.

In older children, particularly those older than 4 years, ad hoc or semi-annual monitoring, that is, twice-yearly dried blood spot testing between plasma-based assessments, may increase the sense of safety among parents and caregivers with regard to self-monitoring. This may, in turn, contribute to reducing the risk of early and late metabolic complications, including the risk of metabolic decompensation.

The findings of this preliminary study support the clinical utility of dried blood spot (DBS)-based monitoring as a practical tool for the dietary management of selected inborn errors of amino acid metabolism. In particular, this approach appears to facilitate timely dietary adjustments in clinically urgent situations, support the correction of metabolic imbalances, and improve the well-being of patients and their families by increasing their sense of safety, autonomy, and active participation in disease management. Importantly, the implementation of DBS-based treatment monitoring may also have broader health-economic implications. Compared with conventional plasma-based testing, DBS sampling has the potential to reduce healthcare system costs by simplifying sample collection, handling, and transport. In addition, home-based or locally performed DBS collection may decrease the financial and logistical burden on families by limiting the need for repeated travel to specialized metabolic centers for follow-up assessments.

Based on the preliminary outcomes of the pilot study and the observed feasibility of tandem mass spectrometry (MS/MS)-based DBS analysis, we propose a list of inborn errors of amino acid metabolism for which this strategy may be particularly suitable for dietary treatment monitoring. The recommended follow-up intervals and proposed biochemical parameters for assessment are summarized in Table 4.

However, this study has several limitations. First, it was a single-center pilot study including small and heterogeneous groups of patients with different inherited disorders of amino acid metabolism. Second, the study did not include a parallel control group monitored exclusively by conventional venous plasma sampling. Third, adherence to scheduled monthly DBS sampling varied between years and disease groups, and additional clinically indicated samples were collected in selected patients, which may have influenced completion-rate calculations. Finally, DBS-derived concentrations were interpreted using adopted reference values corresponding to plasma concentrations; therefore, further validation studies are needed to define disorder- and analyte-specific DBS reference ranges for long-term monitoring.

## 5. Conclusions

Patients with inborn errors of amino acid metabolism should undergo regular in-person medical and dietary follow-up, irrespective of the method used to monitor treatment. Beyond the age specified in the table, dried blood spot-based blood monitoring may be used in any patient on an ad hoc basis, according to current clinical need.

The implementation of tandem MS-based monitoring of dietary treatment in patients with disorders of amino acid metabolism may facilitate the broader adoption of this approach across metabolic centers not only in Poland.

## Figures and Tables

**Table 1:** List of inborn errors of metabolism and the number of patients included in the study in each year.

Inborn Errors of Metabolism	Age Range *	Phase 1		Phase 2	
		No. of Patients in 2022	No. of Patients in 2023	No. of Patients in 2024	No. of Patients in 2025
MSUD	5 months–20.5 years	6	6	7	7
TYR type III	4 months	1	1	1	1
HCU	1–8 years	n.i.	4	4	4
GA1	5 months–7 years	n.i.	12	13	13
MMA	1–9 years	5	5	excl.	excl.
IVA	1–12 years	8	7	excl.	excl.
ASA	12 months	1	1	excl.	excl.
MAT I/III deficiency	2.5 years	1	1	excl.	excl.
BKT deficiency	5 years	1	1	excl.	excl.
PA	6 months	n.i.	1	excl.	excl.
Gyrate atrophy	9 years	n.i.	1	excl.	excl.
Citrullinemia type 1	6.5 years	1	-	excl.	excl.
Total		24	40	25	25

* Age at study enrollment; MSUD, maple syrup urine disease; TYR type III, tyrosinemia type III; HCU, homocystinuria; GA1, glutaric aciduria type 1; MMA, methylmalonic aciduria; IVA, isovaleric aciduria; ASA, argininosuccinic aciduria; MAT I/III deficiency, methionine adenosyltransferase I/III deficiency; BKT deficiency, beta-ketothiolase deficiency; PA, propionic aciduria; n.i., not included in the project; excl., excluded from the project (DBS monitoring discontinued per protocol).

**Table 2:** Completion rate of scheduled dried blood spot (DBS) analyses in individual disease groups during the observation period.

IEM	Patient Age Range	No. of Patients in 2022	No. of DBS Cards Planned/Returned	Completion Rate, %	No. of Patients in 2023	No. of DBS Cards Planned/Returned	Completion Rate, %	No. of Patients in 2024	No. of DBS Cards Planned/Returned	Completion Rate, %	No. of Patients in 2025	No. of DBS Cards Planned/Returned	Completion Rate, %
MSUD	0.5–20.5 years	6	72/70	97	6	72/63	87.5	7	24/24	100	7	24/39	162.5 *
TYR type III	4 months	1	12/12	100	1	12/12	100	1	12/8	66.6	1	12/8	67
HCU	1–8 years	n.i.	n.i.	n.i.	4	48/40	83.3	4	48/9	18.7	4	48/14	29
GA1	1–7 years	n.i.	n.i.	n.i.	12	144/86	59.7	13	156/65	41.6	13	156/54	34.6
MMA	1–12.5 years	5	60/54	90	5	60/44	73.3	excl.	excl.	excl.	excl.	excl.	excl.
IVA	1–12 years	8	96/60	62	7	84/45	53.5	excl.	excl.	excl.	excl.	excl.	excl.
ASA	12 months	1	12/3	25	1	12/8	66.6	excl.	excl.	excl.	excl.	excl.	excl.
MAT I/III deficiency	2.5 years	1	12/3	25	1	12/7	58.3	excl.	excl.	excl.	excl.	excl.	excl.
BKT deficiency	5 years	1	12/11	91.6	1	12/9	75	excl.	excl.	excl.	excl.	excl.	excl.
PA	6 months	-	-	-	1	12/7	58.3	excl.	excl.	excl.	excl.	excl.	excl.
Gyrate atrophy	9 years	-	-	-	1	12/9	75	excl.	excl.	excl.	excl.	excl.	excl.
Citrullinemia type 1	6.5 years	1	12/10	83.3	-	-	-	excl.	excl.	excl.	excl.	excl.	excl.
Total		24	288/223	Average 71.7	40	480/330	Average 71.8	25	240/106	Average 56.7	25	240/115	Average 73

IEM, inborn error of metabolism; MSUD, maple syrup urine disease; TYR type III, tyrosinemia type III; HCU, homocystinuria; GA1, glutaric aciduria type 1; MMA, methylmalonic aciduria; IVA, isovaleric aciduria; ASA, argininosuccinic aciduria; MAT I/III deficiency, methionine adenosyltransferase I/III deficiency; BKT deficiency, beta-ketothiolase deficiency; PA, propionic aciduria; DBS, dried blood spot; n.i., not included in the project; excl., excluded from the project (DBS monitoring discontinued per protocol); * percentages above 100% indicate additional samples submitted and analyzed beyond the initial study plan. In the first year after the inclusion of patients with HCU and GA1 in the project, the completion rates for dried blood spot-based measurements were highest compared with subsequent years of follow-up, reaching approximately 84% and 60%, respectively.

**Table 3:** The number of dietary interventions resulting from abnormal dried blood spot-based results in individual patients during the observation period.

IEM	Patient Age/Sex	No. of DIs in 2022	No. of DIs in 2023	No. of DIs in 2024	No. of DIs in 2025
MSUD	7.5 years/m	4	2	2	1
MSUD	6.5 years/m	3	1	4	2
MSUD	18 years/f	5	3	0	3
MSUD	20.5 years/f	8	6	3	2
MSUD	5 years/f	2	3	1	0
MSUD	2.5 years/f	4	0	LTx	-
MSUD	18 years/m	-	-	0	4
MSUD	6 months/m	-	-	3	2
	Total No.	26	15	13	14
TYR type III	4 months/m	4	4	0	2
HCU	1 year/f	n.i.	1	0	1
HCU	8 years/m		1	0	2
HCU	2.5 years/f		0	-	-
HCU	8 months/f		3	0	0
HCU	6 months/f		-	3	5
	Total No.		5	3	8
GA1	1 year/m	n.i.	1	4	2
GA1	6 months/f		-	2	3
GA1	8 years/m		1	0	0
GA1	5.5 years/m		1	1	0
GA1	8 years/f		1	0	0
GA1	3.5 years/m		1	1	3
GA1	7.5 years/m		1	0	0
GA1	2 years/m		1	1	2
GA1	2.5 years/m		0	0	0
GA1	4.5 years/f		0	0	1
GA1	6 months/m		0	3	6
GA1	5 years/m		1	0	0
GA1	2 years/m		1	0	4
	Total No.		9	12	21
MMA	1 year/m	3	4		
	3 years/m	0	0		
	6.5 years/m	0	0		
	9.5 years/m	0	0		
	12.5 years/m	0	0		
IVA	1 year/m	0	0		
	2 years/m	0	0		
	2.5 years/f	0	0		
	3.0 years/m	0	0		
	3.0 years/f	0	0		
	4.0 years/f	0	0		
	5.5 years/m	0	0		
	12 years/f	0	0		
ASA	12 months/m	0	0		
MAT I/III deficiency	2.5 years/m	3	1		
BKT deficiency	5 years/m	0	0		
PA	6 months/f	-	0		
Gyrate atrophy	9 years/m		0		
Citrullinemia type 1	6.5 years/f	0	-		

IEM, inborn error of metabolism; DIs, dietary interventions; m, male; f, female; MSUD, maple syrup urine disease; TYR type III, tyrosinemia type III; HCU, homocystinuria; GA1, glutaric aciduria type 1; MMA, methylmalonic aciduria; IVA, isovaleric aciduria; ASA, argininosuccinic aciduria; MAT I/III deficiency, methionine adenosyltransferase I/III deficiency; BKT deficiency, beta-ketothiolase deficiency; PA, propionic aciduria; DBS, dried blood spot; n.i., not included in the project; LTx, liver transplantation.

**Table 4:** Proposed disease- and age-specific schedule for DBS-based monitoring of dietary treatment using MS/MS and supplementary biochemical methods.

Inborn Error of Metabolism	Age/Monitoring Schedule	MS/MS and Other Biochemical Parameters
MSUD	Until 7 years of age; monthly	BCAAs
TYR type III	Until 4 years of age; monthly	Tyr, Phe
HCU	Until 4 years of age; monthly	tHcy, Met
GA1	Until 4 years of age; monthly	Lys, Trp
MMA	Until 12 months of age; monthly	MMA, MCA, BCAAs
MAT I/III deficiency, diet-treated	Until 4 years of age; monthly	Met

MSUD—maple syrup urine disease; TYR type III—tyrosinemia type III; HCU—homocystinuria; GA1—glutaric aciduria type 1; MMA—methylmalonic aciduria; MAT I/III deficiency—methionine adenosyltransferase I/III deficiency; MS/MS—tandem mass spectrometry; BCAAs—branched-chain amino acids; Tyr—tyrosine; Phe—phenylalanine; tHcy—total homocysteine; Met—methionine; Lys—lysine; Trp—tryptophan; MCA—methylcitric acid.

## Data Availability

The data presented in this study are available on request from the corresponding author due to restrictions related to clinical data obtained as part of patient care and monitoring, which are subject to restricted access.

## Abbreviations

The following abbreviations are used in this manuscript:

allo-Ile	Alloisoleucine
AR	Abnormal result
ASA	Argininosuccinic aciduria
BCAAs	Branched-chain amino acids
BKT deficiency	Beta-ketothiolase deficiency
DBS	Dried blood spot
DI	Dietary intervention
GA1	Glutaric aciduria type 1
HCU	Homocystinuria
Hcy	Homocysteine
HFBA	Heptafluorobutyric acid
IEM	Inborn error of metabolism
IEMs	Inborn errors of metabolism
Ile	Isoleucine
IVA	Isovaleric aciduria
Leu	Leucine
LTx	Liver transplantation
Lys	Lysine
MAT I/III deficiency	Methionine adenosyltransferase I/III deficiency
MCA	Methylcitric acid
Met	Methionine
MMA	Methylmalonic aciduria
MRM	Multiple reaction monitoring
MS/MS	Tandem mass spectrometry
MSUD	Maple syrup urine disease
PA	Propionic aciduria
Phe	Phenylalanine
PKU	Phenylketonuria
tHcy	Total homocysteine
Trp	Tryptophan
Tyr	Tyrosine
TYR type III	Tyrosinemia type III
Val	Valine
